# The Prognostic Value of Maximum Standardized Uptake Value (SUVmax) of 18F-FDG PET/CT for Risk Stratification and Outcome Prediction in Endometrial Cancer: A Retrospective Analysis

**DOI:** 10.7759/cureus.80109

**Published:** 2025-03-05

**Authors:** Hiromi Shibuya, Yoichi Kobayashi, Momoe Watanabe, Mai Momomura, Hironori Matsumoto, Tohru Morisada

**Affiliations:** 1 Department of Obstetrics and Gynecology, Kyorin University Faculty of Medicine, Tokyo, JPN

**Keywords:** endometrial cancer, pet/ct, prognosis, risk stratification, suvmax

## Abstract

Background and objective

Endometrial cancer, the most common gynecological malignancy in developed countries, is frequently diagnosed at an early stage due to the symptomatic presentation of irregular bleeding. We aimed to assess the prognostic value of preoperative PET/CT-derived maximum standardized uptake value (SUVmax) in patients with endometrial cancer and investigated its association with established clinical risk factors and survival outcomes.

Methods

We retrospectively analyzed 162 patients with endometrial cancer who underwent preoperative PET/CT at Kyorin University Hospital from December 2020 to June 2024. We recorded and assessed the SUVmax of primary uterine tumors against clinical histopathological findings, such as myometrial invasion, lymphovascular space invasion (LVSI), cervical stromal invasion, extrauterine lesions, and histological types. Patients were categorized into recurrence risk groups based on the Japan Society of Gynecologic Oncology (JSGO) 2023 guidelines. Statistical evaluations involved the Mann-Whitney U tests for group comparisons and Kaplan-Meier analysis for overall survival (OS) and progression-free survival (PFS).

Results

The median SUVmax of primary uterine tumors was 26.0. A higher SUVmax was significantly associated with myometrial invasion, LVSI, and larger tumor diameter (p<0.01). Significant differences in the SUVmax were observed among the various postoperative recurrence risk groups, with higher values observed in the intermediate- and high-risk groups (p<0.01). Kaplan-Meier survival analysis revealed that a higher SUVmax was significantly associated with poorer OS.

Conclusions

SUVmax derived from preoperative PET/CT can significantly predict endometrial cancer prognosis, correlating with clinical risk factors and OS. Hence, integrating SUVmax into preoperative assessments could enhance risk stratification and inform treatment planning. Prospective studies with larger cohorts are needed to validate these findings and establish standardized SUVmax cutoffs for clinical application.

## Introduction

Endometrial cancer is the most common gynecological malignancy in developed countries, and its incidence is on the rise [1]. It is often diagnosed at stage I [2] due to its early symptom presentation, primarily irregular bleeding. Endometrial cancer arises from the glandular epithelium of the endometrium and was traditionally classified into estrogen-dependent type I and estrogen-independent type II tumors. Specifically, type I develops after endometrial hyperplasia and is generally low-grade with a favorable prognosis, as it does not easily metastasize. In contrast, type II is a poorly differentiated endometrial carcinoma or a histological type other than endometrial carcinoma (e.g., grade 3, serous carcinoma, and clear cell carcinoma). It arises de novo from the atrophic endometrium of elderly patients and is associated with a poor prognosis [3-5]. Endometrial cancer is classified into low-, intermediate-, and high-risk categories based on postoperative pathological findings. This classification is crucial as it determines the likelihood of recurrence and the need for adjuvant therapies, such as chemotherapy.

Unlike cervical cancer, endometrial cancer is less radiosensitive [6], and chemotherapy is less effective compared to ovarian cancer. Consequently, surgery remains the primary treatment option for endometrial cancer. Preoperative staging is essential for establishing recurrence risk groups by assessing myometrial and cervical invasion and lymph node metastasis, thereby providing critical information for surgical management. The 2025 edition of the National Comprehensive Cancer Network (NCCN) Guidelines recommends contrast-enhanced MRI for evaluating local extension, including deep myometrial invasion and cervical stromal invasion [7]. However, one study has reported that myometrial involvement greater than 50% was underestimated in 21% of grade 1 endometrioid adenocarcinoma cases and 32% of grade 2 adenocarcinoma cases when using MRI [8]. Although MRI is a valuable tool for preoperative staging of endometrial cancer, its limitations highlight the need for complementary diagnostic approaches.

18F-fluorodeoxyglucose (FDG) PET/CT is a valuable modality for preoperative staging, treatment planning, and recurrence detection. It is particularly versatile in diagnosing gynecological tumors. The standardized uptake value (SUV), a semi-quantitative parameter indicating the degree of FDG uptake, is recognized as a significant marker of tumor aggressiveness and prognosis in various malignancies, including endometrial cancer [9,10]. Notably, noninvasive tests that could be conducted preoperatively to accurately assess and stage endometrial cancer would provide significant advantages for patients.

In recent years, molecular classification has been increasingly recognized as an important prognostic and predictive factor in endometrial cancer. The latest International Federation of Gynecology and Obstetrics (FIGO) classification incorporates The Cancer Genome Atlas (TCGA) classification, which categorizes endometrial cancer into four distinct molecular subtypes. Recent studies suggest that these molecular subtypes have a significant impact on prognosis and treatment response, independent of conventional clinicopathological factors [11]. However, in Japan, the clinical implementation of molecular subtyping remains limited due to resource constraints and the lack of standardized protocols. As a result, our study did not incorporate molecular subtyping as a parameter in the current analysis.

In this study, we aimed to investigate the relationship between maximum SUV (SUVmax) and other clinical risk factors for recurrence in patients with endometrial cancer undergoing preoperative PET/CT. Additionally, we aimed to evaluate whether the noninvasive measurement of SUVmax using PET/CT could serve as a reliable predictor of prognosis. We hypothesize that higher SUVmax values are significantly associated with more aggressive tumor characteristics, including higher tumor grade, deeper myometrial invasion, and increased lymphovascular space invasion (LVSI). Furthermore, we anticipate that elevated SUVmax will correlate with poorer overall survival (OS) and progression-free survival (PFS), making it a potential independent prognostic biomarker for endometrial cancer.

## Materials and methods

A total of 191 patients were diagnosed with endometrial cancer between December 2020 and June 2024 at Kyorin University Hospital (Tokyo, Japan). Of these, 162 patients who underwent preoperative PET/CT were included in this study. Exclusions were made for the following reasons: six patients received neoadjuvant chemotherapy, four patients had concurrent cancers (e.g., cervical cancer, ovarian cancer, and malignant lymphoma), and two patients showed no FDG accumulation on PET/CT. Additionally, 17 patients who did not undergo PET/CT preoperatively, six patients without a pathological diagnosis, two patients diagnosed with endometrial cancer postoperatively after an initial diagnosis of a benign tumor, and five patients with poorly controlled diabetes (blood glucose levels >150 mg/dL), which could affect FDG uptake, were also excluded. Furthermore, one patient could not undergo an outpatient PET/CT due to hospitalization, and another refused to undergo a PET/CT examination.

**Figure 1:**
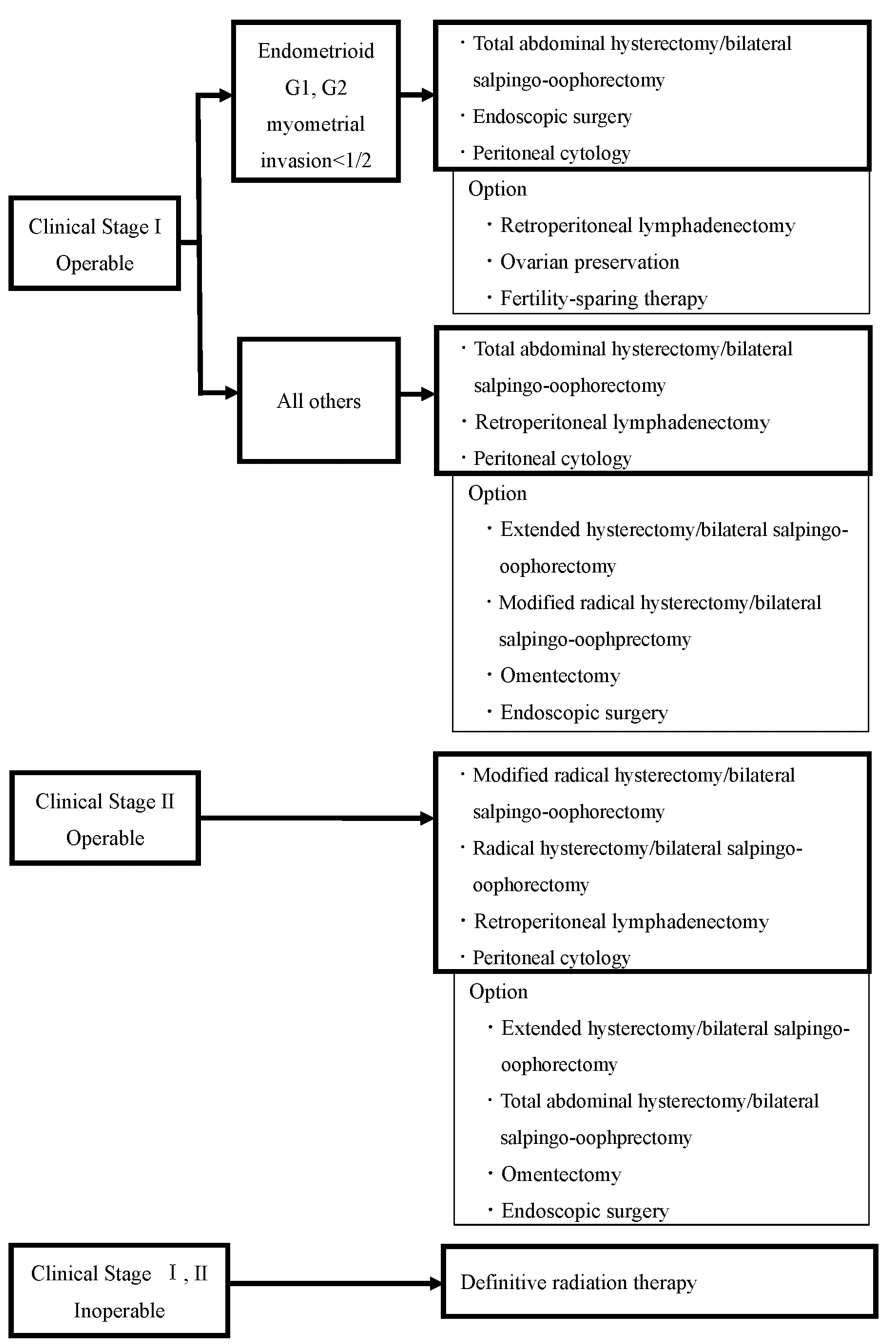
The initial treatment for patients with endometrial cancer preoperatively considered as stage I or II Modified from the Japan Society of Gynecologic Oncology 2023 guidelines for the treatment of uterine body neoplasms [12]

Following an endometrial biopsy confirming the diagnosis of endometrial cancer, all patients underwent MRI and PET/CT preoperatively. The preoperative staging was determined based on imaging findings. Surgical procedures included laparotomy, laparoscopic or robot-assisted laparoscopic hysterectomy, bilateral salpingo-oophorectomy, and pelvic lymphadenectomy. Lymph node dissection was performed following the Japan Society of Gynecologic Oncology (JSGO) 2023 guidelines for uterine body neoplasms [12], except in low-risk patients (stage I, endometrioid carcinoma Grade 1 or Grade 2 with less than half myometrial invasion) or those who declined lymph node dissection (Figure1). Sixty patients did not undergo lymph node dissection. 

The SUVmax of primary uterine tumors and risk factors for recurrence, including histological type, stage, myometrial invasion, LVSI, cervical stromal invasion, and extrauterine lesions, as well as prognosis, were retrospectively analyzed.

This study was approved by the Institutional Review Board of Kyorin University (approval no. 1977).

18F-FDG PET/CT

PET/CT was performed using a Cartesion Prime scanner (Canon Medical Systems, Tochigi, Japan), covering the region from the crown of the head to the mid-thigh. The CT parameters for attenuation correction were as follows - tube voltage: 120 kV; field of view: 0.813; helical pitch: 65; and slice thickness: 2 mm. Patients with blood glucose levels <150 mg/dL were eligible for inclusion, while those with higher levels required consultation with their attending physician before the procedure. Each patient received an FDG injection at a dose of 3.7 MBq/kg. After a one-hour resting period, PET images were acquired with a matrix size of 336 × 336, with 180 s per bed position. Whole-body imaging was performed in six bed positions, while the abdominal and pelvic regions were imaged in three bed positions, with each position captured for three minutes.

The SUVmax of primary tumors was measured by a single physician who is both a radiologist and a nuclear medicine specialist. The regions of interest were manually defined according to a standardized protocol, and all measurements were conducted by the same specialist to ensure consistency.

Survival data and statistical analysis

The SUVmax between groups was compared using the Mann-Whitney U test. This test was selected because SUVmax and other clinical parameters did not follow a normal distribution and contained outliers. As a non-parametric method, it is more robust to skewed data and extreme values compared to the t-test, making it a suitable choice for this analysis. OS was defined as the duration from the date of surgery to either the date of death due to endometrial cancer or the last follow-up observation. PFS was defined as the time from surgery to the date of recurrence. Survival curves were generated using the Kaplan-Meier method. The median follow-up period was 24 (range 5-50) months.

## Results

Patient characteristics

The patient characteristics are summarized in Table 1. The median age was 56.5 (range: 34-86) years, and the median SUVmax of the uterine tumors was 26.0 (range: 5.7-124.9). MRI failed to detect tumors in three patients, while measurable tumors were identified in 159 patients, with a median tumor size of 3.7 (range: 0.6-13) cm. In this study, MRI-based tumor size and depth of invasion were included in the analysis. Therefore, for the three cases where MRI failed to detect the tumor, the corresponding data were considered missing and these cases were excluded from the relevant analysis. Among the patients, 103 were postmenopausal, and 59 were premenopausal. The median cancer antigen (CA) 125 level was 21.2 (range: 3.6-1325) U/mL and the median BMI was 23.7 (range: 16.8-49.6) kg/m². Surgical approaches included laparotomy in 93 patients and laparoscopic surgery in 69, with lymph node dissection performed in 102 patients. When comparing maximum tumor diameters, the median SUVmax was 23.4 in 80 patients with a tumor diameter <3.7 cm and 29.2 in 82 patients with a tumor diameter ≥3.7 cm, demonstrating a significant difference (p<0.05).

**Table 1:** Patient characteristics (N=162) BMI: body mass index; CA: cancer antigen; FIGO: International Federation of Gynecology and Obstetrics; SUVmax: maximum standardized uptake value

Variables	Values
	Median (range)
Age, years	56.5 (34–86)
SUVmax	26.0 (5.7–124.9)
Tumor maximum size, cm	3.7 (0.6–13)
CA 125	21.2 (3.6–1325)
BMI, kg/m^2^	23.7 (16.8–49.6)
Menopause	Number (%)
Postmenopause	103 (63.6)
Premenopause	59 (36.4)
FIGO stage	Number (%)
IA	101 (62.3)
IB	22 (13.6)
Ⅱ	11 (6.8)
IIIA	5 (3.1)
IIIB	5 (3.1)
IIIC1	9 (5.6)
IIIC2	3 (1.8)
ⅣB	6 (3.7)
Histology	Number (%)
Endometrioid carcinoma G1	106 (65.4)
Endometrioid carcinoma G2	17 (10.5)
Endometrioid carcinoma G3	10 (6.2)
Carcinosarcoma	10 (6.2)
Mixed	6 (3.7)
Dedifferentiated carcinoma	3 (1.8)
Serous carcinoma	3 (1.8)
Clear cell carcinoma	3 (1.8)
Other	4 (2.5)

FIGO staging of the cohort revealed 101 patients with stage IA, 22 with stage IB, 11 with stage II, five with stage IIIA, five with stage IIIB, nine with stage IIIC1, three with stage IIIC2, and six with stage IVB. Differences in SUVmax according to FIGO stage and histological type are shown in Table 2.

**Table 2:** Differences in SUVmax according to tumor stage and histological type *G1 vs. G2: p<0.05. **G1 vs. G3: p<0.05 FIGO: International Federation of Gynecology and Obstetrics; SUVmax: maximum standardized uptake value

FIGO stage	Number (%)	SUVmax median	P-value
Ⅰ	123 (75.9)	25.2	NS
Ⅱ	11 (6.8)	26.0	
Ⅲ	22 (13.6)	29.2	
Ⅳ	6 (3.7)	27.0	
FIGO Stage			
IA	101 (62.3)	23.5	0.077
≥ⅠB	61 (37.7)	27.6	
FIGO Stage			
Ⅰ–Ⅱ	134 (82.7)	25.4	0.93
Ⅲ–Ⅳ	28 (17.3)	29.4	
Histology			
G1	106 (65.4)	25.1	0.045*
G2	17 (10.5)	27	
G3	10 (6.2)	32.6	0.039**
Non-endometrioid	29 (17.9)	27.5	
Histology			
Type Ⅰ (G1+G2)	123 (75.9)	25.3	0.22
Type II (G3+other)	39 (24.1)	28.9	

The median SUVmax by FIGO stage was 25.2 for stage I, 26.0 for stage II, 29.2 for stage III, and 27.0 for stage IV, with no statistically significant differences among the four groups. The median SUVmax by histological grade was 25.1 for grade 1, 27.0 for grade 2, and 32.6 for grade 3, with a significant difference observed between grade 1 and grade 2, as well as between grade 1 and grade 3 (p<0.05). For stage IA patients, the median SUVmax was 23.5 compared to 27.6 for stage IB or higher (p=0.07), showing no statistically significant difference. Similarly, there was no significant difference in the SUVmax between Type I and Type II tumors (p=0.22).

The association between clinicopathological findings and SUVmax is summarized in Table 3. Among the patients, 57 had no myometrial invasion, 54 had less than 1/2 myometrial invasion, and 51 had more than 1/2 myometrial invasion. The mean SUVmax was 21.5 for patients without myometrial invasion, 28.0 for those with less than 1/2 myometrial invasion, and 28.9 for those with more than 1/2 myometrial invasion. A significant difference in the SUVmax was observed between patients without myometrial invasion and those with any degree of myometrial invasion (p<0.01). Furthermore, significant differences were noted between cases without myometrial invasion and those with <1/2 myometrial invasion (p<0.01) and between cases without myometrial invasion and those with ≥1/2 myometrial invasion (p<0.05).

**Table 3:** Association between SUVmax and clinicopathological characteristics *P<0.05. **P<0.01 LVSI: lymphovascular space invasion; SUVmax: maximum standardized uptake value

Variables	Values		
Myometrial invasion	Number (%)	SUVmax median	P-value
No invasion	57 (35.2)	21.5	No vs. ≥1/2: 0.011*
<1/2	54 (33.3)	28	No vs. <1/2: 0.008**
≧1/2	51 (31.5)	28.9	No vs. MI+: 0.003**
LVSI			
Negative	110 (67.9)	25.1	0.018*
Positive	52 (32.1)	29.6	
Cervical involvement			
Negative	141 (87.0)	25.5	0.38
Positive	21 (13.0)	27	
Lymph node metastasis			
Negative	83 (51.2)	31.1	0.083
Positive	19 (11.7)	27.5	
No lymphadenectomy	60 (37.0)	20	
Extrauterine lesion			
Negative	136 (84.0)	25.4	0.32
Positive	26 (16.0)	29	
Peritoneal cytology			
Negative	149 (92.0)	26	0.95
Positive	13 (8.0)	28.9	
Tumor maximum size			
<3.7 cm	80 (49.4)	23.4	0.023*
≥3.7 cm	82 (50.6)	29.2	
Risk			
Low	83 (51.2)	23.5	L vs. I: p=0.05
Intermediate	26 (16.0)	29.2	L vs. H: p=0.043*
High	53 (32.7)	28.2	I vs. H: p=0.49
Risk			
Low	83 (51.2)	23.5	0.014*
Intermediate/high	79 (48.8)	28.6	

The mean SUVmax was 23.5 in 110 patients without LVSI and 29.6 in 52 patients with LVSI, showing a significant difference (p<0.05). For cervical stromal invasion, the mean SUVmax was 25.5 in 141 patients without invasion and 27.0 in 21 patients with invasion, although this difference was not statistically significant (p=0.38). Similarly, the mean SUVmax was 31.1 in 83 patients without lymph node metastasis and 27.5 in 19 patients with lymph node metastasis (p=0.08). For ascites cytology, the mean SUVmax was 26.0 in 149 patients with negative results and 28.9 in 13 patients with positive results (p=0.94). Additionally, the mean SUVmax was 25.4 in 136 patients without extrauterine lesions and 28.9 in 25 patients with extrauterine lesions (p=0.32). When comparing maximum tumor diameters, the median SUVmax was 23.4 in 80 patients with a tumor diameter <3.7 cm and 29.2 in 82 patients with a tumor diameter ≥3.7 cm, demonstrating a significant difference (p<0.05).

Postoperative recurrence risk classification

**Figure 2:**
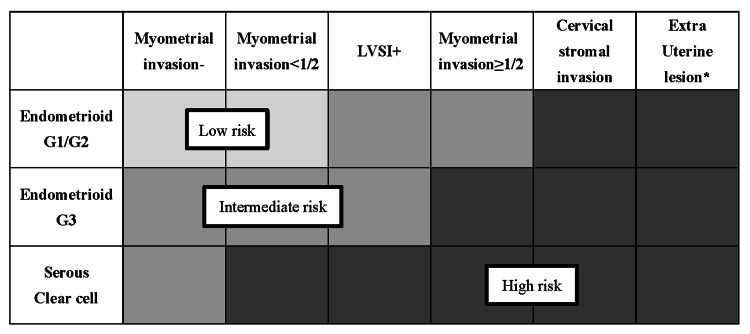
Classification of postoperative risk of endometrial cancer *Extrauterine lesions: adnexa, uterine serosa, vagina, cardinal ligament, lymph node, bladder, rectum, intraperitoneal, and distant metastasis Modified from the Japan Society of Gynecologic Oncology 2023 guidelines for the treatment of uterine body neoplasms [12] LVSI: lymphovascular space invasion

All patients were classified according to the postoperative recurrence risk categories outlined in the JSGO 2023 guidelines for uterine body neoplasms (Figure 2) [12]. The distribution of patients across the postoperative recurrence risk groups was as follows: 83 in the low-risk group, 26 in the intermediate-risk group, and 53 in the high-risk group (Table 3). The median SUVmax values for these groups were 23.2, 29.2, and 28.2, respectively. A significant difference in SUVmax was observed between the low- and high-risk groups (p<0.05). Additionally, when comparing the low-risk group with the combined intermediate-to-high-risk groups, the median SUVmax values were 23.2 and 28.6, respectively, which also showed a significant difference (p<0.05).

Correlation of postoperative recurrence risk classification with patient survival

**Figure 3:**
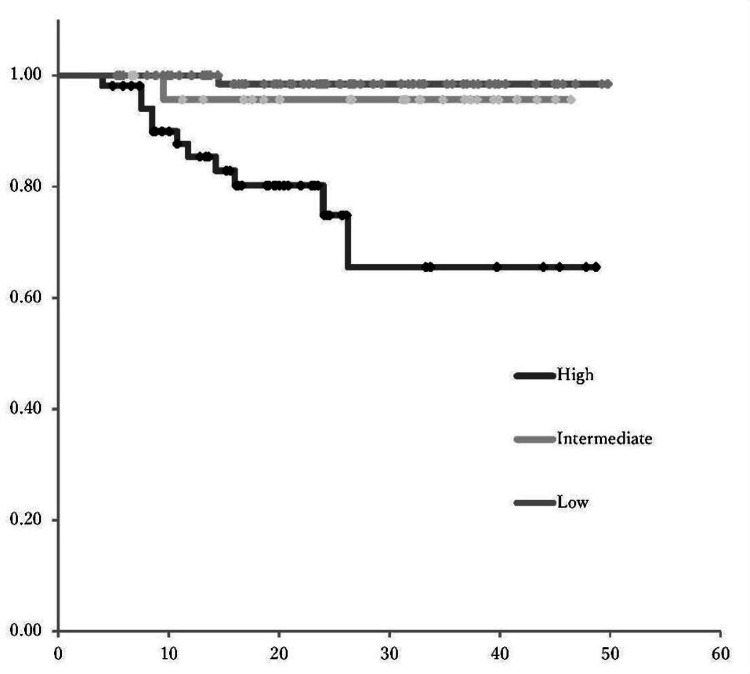
Risk-based PFS Kaplan-Meier survival analysis showed significantly lower PFS in high-risk patients compared to low- and intermediate-risk groups (p<0.001, p<0.05) PFS: progression-free survival

**Figure 4:**
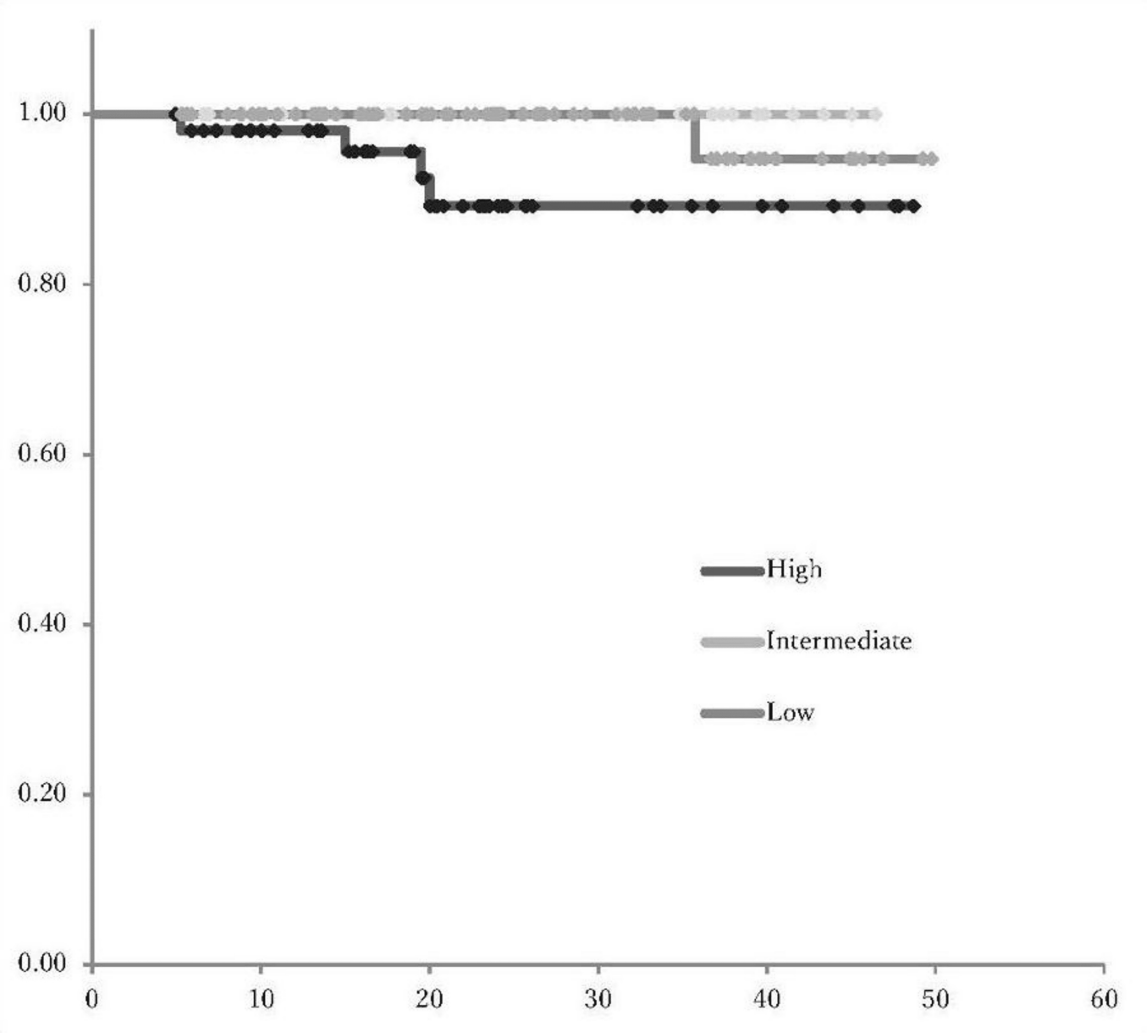
Risk-based overall survival Kaplan-Meier survival analysis showed significantly lower overall survival in high-risk patients compared to low-risk groups (p<0.05)

This study compared the PFS and OS rates among the three groups: low-risk, intermediate-risk, and high-risk (Figures 3, 4). The PFS in high-risk patients was significantly lower than that in the low- and intermediate-risk groups (p<0.001). Similarly, the OS was significantly lower in the high-risk group compared to both the low- and intermediate-risk groups (p<0.05).

Determining the cutoff value for SUVmax

**Figure 5:**
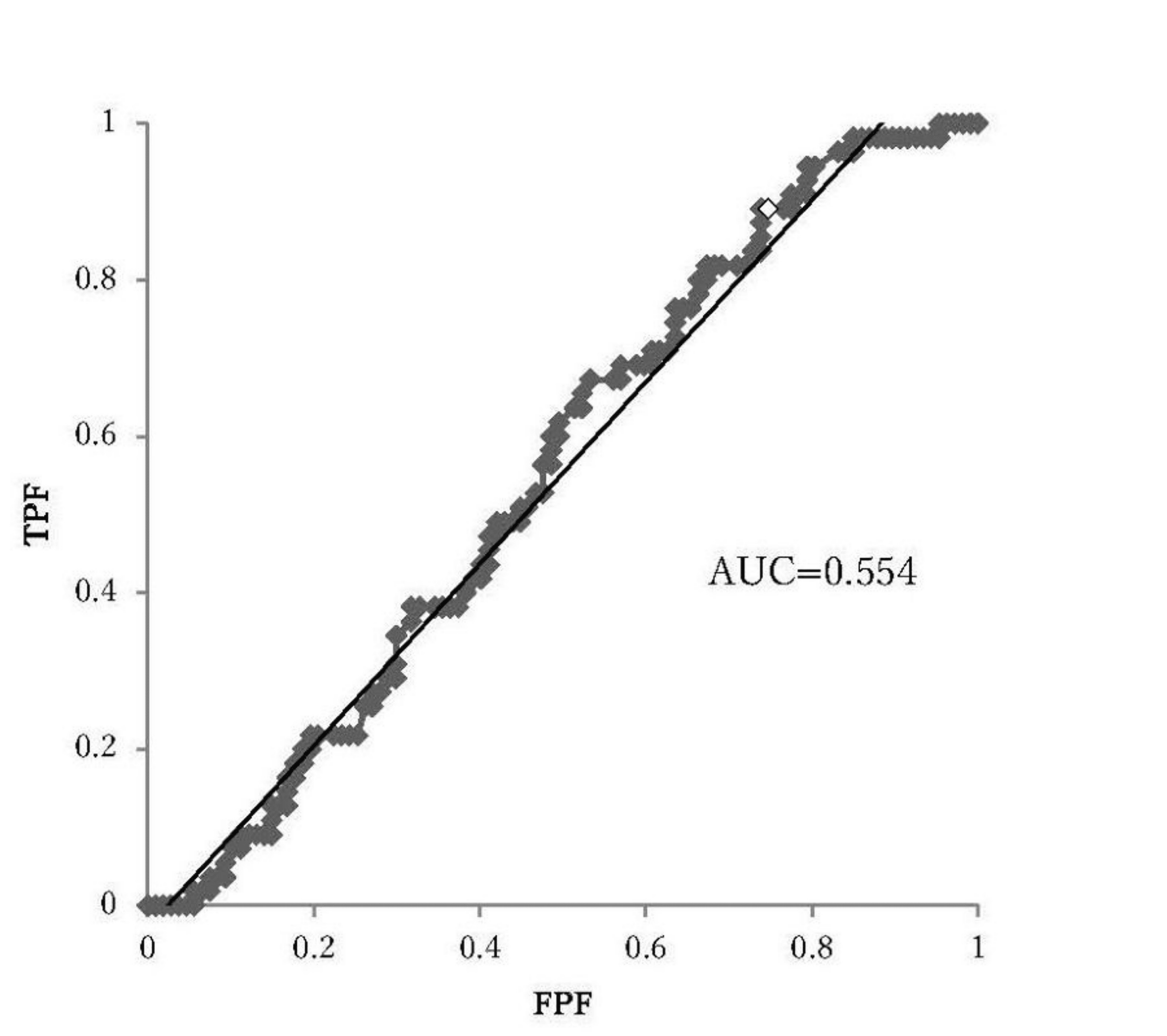
Receiver operating characteristic curve analysis of the SUVmax performed to determine the cutoff values for predicting the presence of risk factors AUC: area under the curve; FPF: false positive fraction; SUVmax: maximum standardized uptake value; TPF: true positive fraction

**Figure 6:**
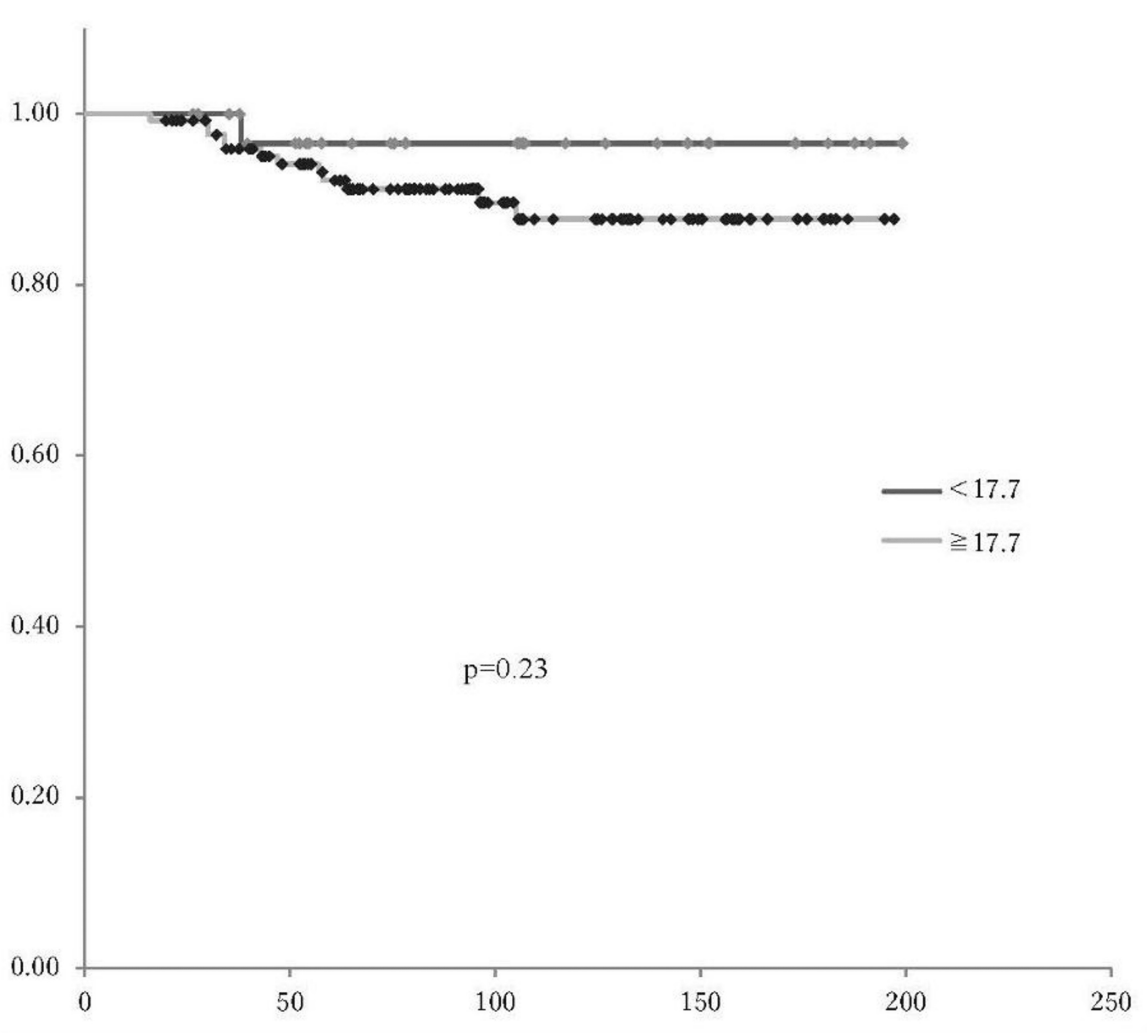
PFS based on SUVmax cutoff of 17.7 Kaplan-Meier survival curves showed no statistically significant difference in PFS between the two groups (p=0.23) PFS: progression-free survival; SUVmax: maximum standardized uptake value

**Figure 7:**
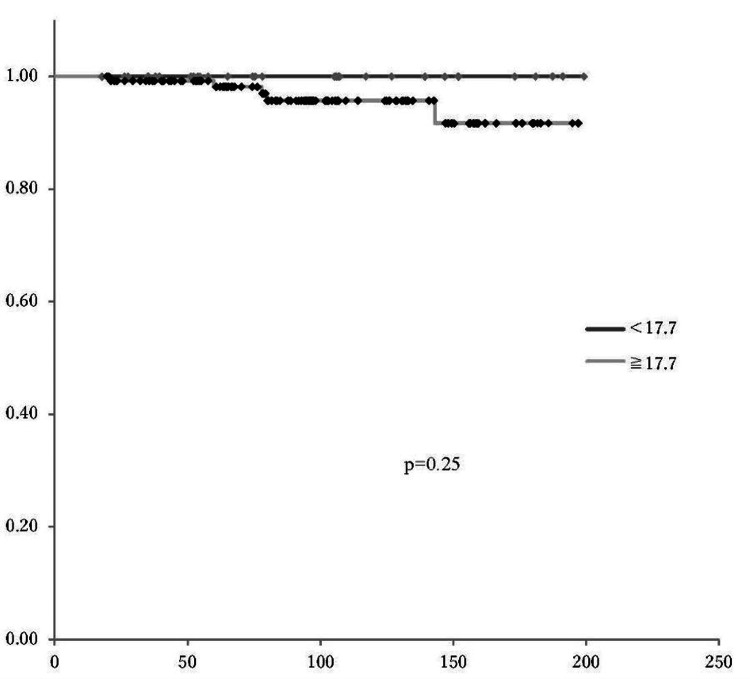
Overall survival based on an SUVmax cutoff of 17.7 Kaplan-Meier survival curves showed no statistically significant difference in overall survival between the two groups (p=0.25) SUVmax: maximum standardized uptake value

**Figure 8:**
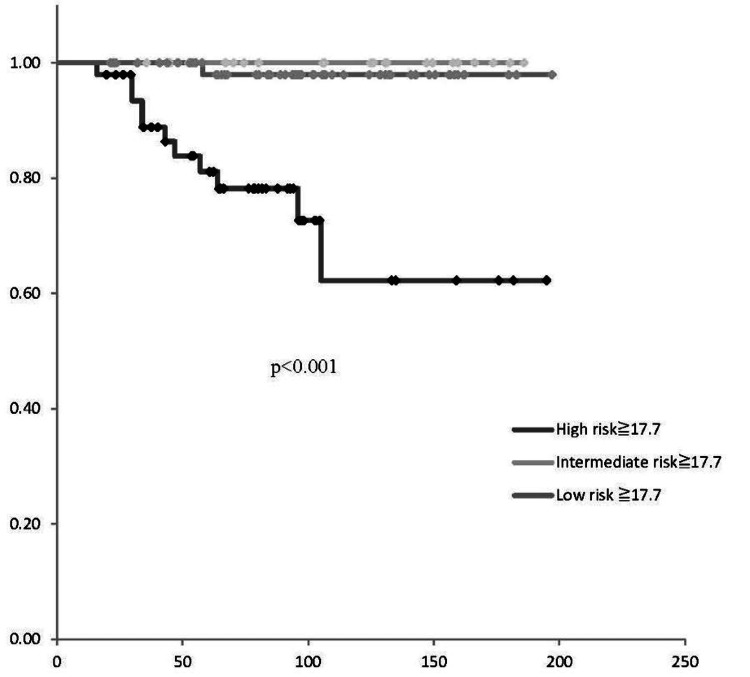
Risk-based PFS based on SUVmax cutoff of 17.7 A significant difference in PFS was noted among the risk groups (p<0.001), with high-risk patients exhibiting poorer progression-free survival compared to intermediate and low-risk groups PFS: progression-free survival; SUVmax: maximum standardized uptake value

**Figure 9:**
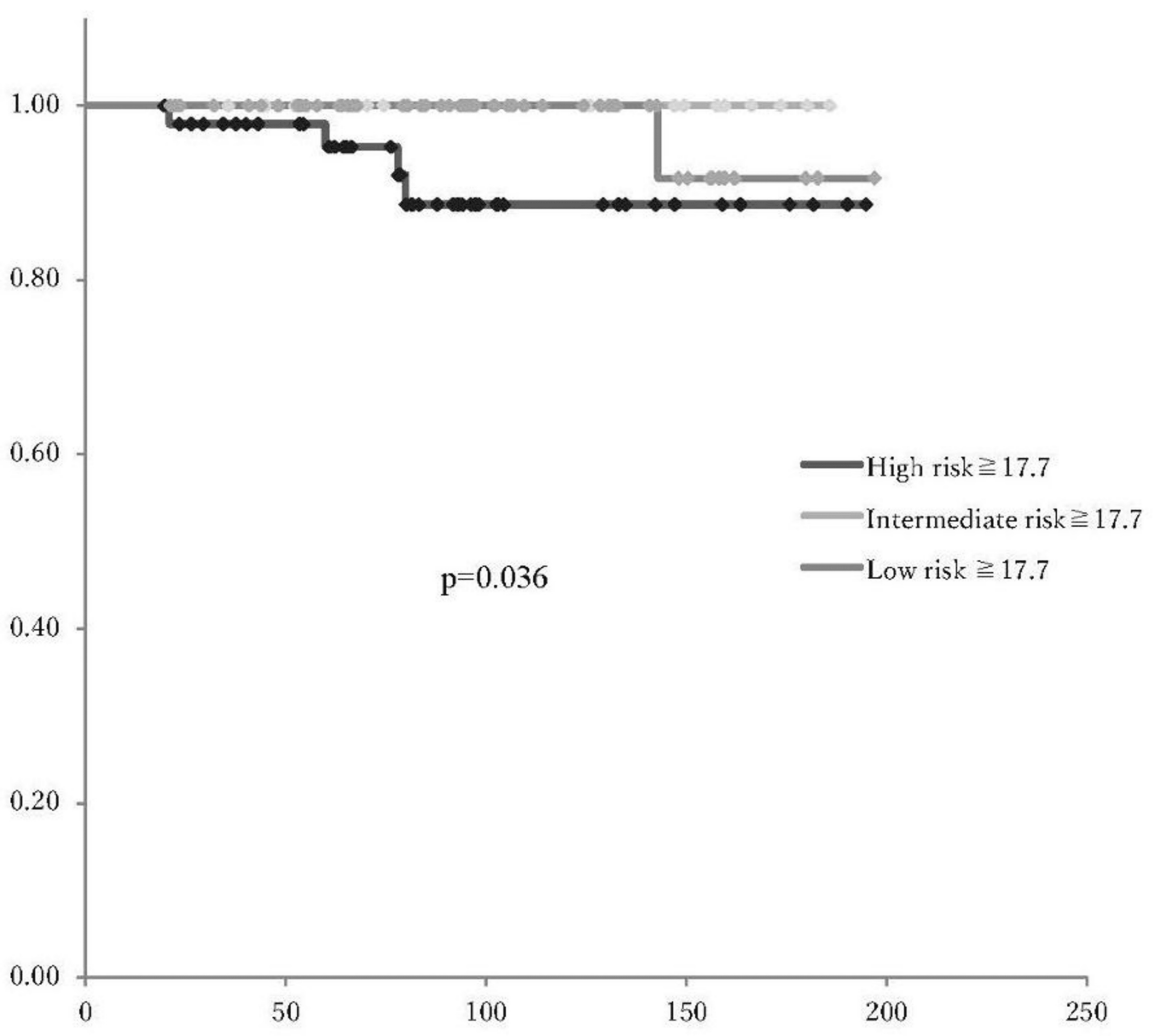
Risk-based overall survival based on SUVmax cutoff of 17.7 A significant difference in overall survival was noted among the risk groups (p=0.036), with high-risk patients exhibiting poorer progression-free survival compared to intermediate and low-risk groups SUVmax: maximum standardized uptake value

The optimal cutoff value of SUVmax for predicting OS and PFS was determined to be 17.7, based on receiver operating characteristic (ROC) curve analysis (Figure 5). There was no significant difference in PFS and OS according to the SUVmax cutoff value (p=0.23, p=0.25, respectively) (Figures 6, 7). In patients with SUVmax >17.7 across the three recurrence risk groups (low-risk, intermediate-risk, and high-risk), PFS and OS were compared. The high-risk group exhibited a significantly poorer prognosis (Figures 8, 9).

## Discussion

MRI is regarded as the most efficient imaging modality for preoperative staging of endometrial cancer, offering a high interobserver agreement rate [13,14]. A meta-analysis of 47 studies demonstrated that contrast-enhanced MRI is significantly more effective than standard MRI and transvaginal ultrasound for assessing myometrial invasion, and it tends to outperform CT [14]. However, preoperative evaluation using MRI and CT alone often provides incomplete risk assessment for endometrial cancer [15,16]. Consequently, we aimed to investigate the additional utility of SUVmax measured by PET/CT alongside conventional MRI evaluation.

Several studies have explored the relationship between preoperative SUVmax values on PET/CT and prognostic factors in endometrial cancer [9,17-29]. Among these, a study conducted by Antonsen et al. [17] was the only one to include over 200 patients, although it also encompassed cases of atypical endometrial hyperplasia. In contrast, our study exclusively focused on patients with endometrial cancer, highlighting the potential of SUVmax as a prognostic marker specifically in this population.

This study assessed whether SUVmax measured by PET/CT is as effective as conventional prognostic factors - such as myometrial invasion, cervical invasion, LVSI, and extrauterine extension - in predicting prognosis in patients with endometrial cancer. Among the 162 patients analyzed, the median age was 56.5 years, and the median SUVmax of tumors was 26.0. A key finding was the significant association between SUVmax and several clinicopathological features, including the extent of myometrial invasion, the presence of LVSI, and tumor diameter. Furthermore, SUVmax differed significantly across postoperative recurrence risk classifications and correlated with both PFS and OS, particularly within high-risk groups.

Our results align with those of previous studies supporting the prognostic utility of PET/CT and SUVmax in endometrial cancer [9,10,20-23]. Prior research has consistently shown that higher SUVmax values are associated with poorer outcomes and advanced disease stages [19,23,24], emphasizing their role as markers of tumor aggressiveness​ [10,15,19,24]. The significant differences in SUVmax between the low-risk and intermediate-risk groups, as well as between low-risk and combined intermediate-to-high-risk groups, further demonstrate that SUVmax is linked to survival outcomes and recurrence risk [15,20,23,25,26].

The observed relationship between higher SUVmax values and worse prognostic factors may reflect the increased metabolic activity and aggressiveness of tumors with elevated FDG uptake [27]. Tumors exhibiting deeper myometrial invasion, LVSI, and larger diameters tend to have higher glycolytic activity, as evidenced by elevated SUVmax. This hypermetabolic state indicates more aggressive tumor behavior and a higher likelihood of metastasis and recurrence, providing a rationale for its association with worse prognostic outcomes.

As shown in Table 3, the median SUVmax was significantly higher in tumors with greater myometrial invasion, the presence of LVSI, and larger tumor diameters. These findings are consistent with those of previous studies demonstrating correlations between elevated SUVmax and aggressive tumor characteristics in endometrial cancer [7,14,18-21]. Specifically, the increased SUVmax in patients with deeper myometrial invasion and LVSI suggests that SUVmax could serve as an indicator of tumor invasiveness and metastatic potential. Nakamura et al. also reported that the preoperative SUVmax of the primary tumor in 44 patients with endometrial cancer was significantly correlated with histological grade and the maximum tumor size [10]. In our study, significant differences in the SUVmax were observed between G1 and G2, as well as between G1 and G3, in patients with endometrioid carcinoma.

Furthermore, previous studies have demonstrated that SUVmax is a significant predictor of OS in various malignancies, including endometrial cancer [17, 22-24]. However, in our study, with a median SUVmax cutoff of 17.7 determined via ROC curve analysis, no significant differences in SUVmax were observed in relation to PFS and OS. One possible explanation for this lack of correlation is the influence of confounding factors. Heterogeneity in adjuvant treatments, particularly chemotherapy regimens, may have mitigated the impact of the impact of SUVmax on long-term outcomes. Additionally, the relatively short median follow-up period of 24.0 months may have limited the ability to detect significant survival differences. Moreover, the retrospective design of this study introduces inherent limitations, including potential selection bias due to patient inclusion based on PET/CT data availability and missing clinical or molecular information, which may affect the completeness of the analysis. A prospective study with predefined inclusion criteria and comprehensive molecular profiling is needed to further validate our findings.

Our analysis revealed significant differences in SUVmax among the risk groups defined by the JSGO 2023 guidelines [12]. The low-risk group demonstrated a significantly lower median SUVmax compared to the high-risk group, and a significant difference was also observed between the low-risk and combined intermediate-to-high-risk groups. PFS and OS were compared among the three recurrence risk groups using the SUVmax cutoff value of 17.7 derived from the ROC curve. In the high-risk group, both PFS and OS were significantly poorer. These findings suggest that SUVmax can be integrated into existing risk stratification systems to enhance their accuracy in predicting recurrence and survival outcomes.

The potential utility of SUVmax as a biomarker is further supported by its strong correlation with established prognostic factors, such as myometrial invasion, LVSI, and tumor size. Given the limitations of traditional imaging modalities and the increasing need for more precise prognostic tools, incorporating PET/CT and SUVmax measurements into routine clinical practice could provide a more comprehensive approach to preoperative evaluations.

Our findings align with prior research indicating that elevated SUVmax values are associated with poor prognosis in endometrial cancer [19-22]. However, despite the significant associations with clinicopathological factors, we did not observe a strong correlation between SUVmax and PFS or OS at the determined median cutoff of 17.7. This suggests that while SUVmax is a valuable prognostic marker, it may not serve as the sole predictor of long-term survival outcomes. Instead, its prognostic significance is likely enhanced when combined with other clinical and pathological variables.

This study has several limitations, which should be considered when interpreting these results. The retrospective, single-center design limits causality assessment and introduces selection bias, restricting generalizability. The exclusion of certain patient groups and inconsistencies in SUVmax cutoff values across studies further affect applicability. Standardized, multicenter prospective studies are needed to validate our findings, establish a universal SUVmax threshold, and enhance clinical utility. Additionally, incorporating molecular and genetic markers into future research could provide a more comprehensive understanding of prognostic factors in endometrial cancer.

## Conclusions

Based on our findings, SUVmax derived from preoperative PET/CT significantly correlated with prognostic factors and survival outcomes in endometrial cancer. Integrating SUVmax into risk stratification models may improve the accuracy of recurrence prediction and enhance preoperative risk assessment. These findings highlight the potential role of SUVmax in guiding treatment strategies. However, further large-scale prospective studies are necessary to validate these findings and define standardized SUVmax cutoff values for clinical application.
